# Supplementary material for: OsFBN6 Enhances Brown Spot Disease Resistance in Rice
Source: Plants (Basel). 2024 Nov 25;13(23):3302. doi: 10.3390/plants13233302 (PMC11644752; doi:10.3390/plants13233302)
Supplement: Supplementary file 1 [file plants-13-03302-s001.zip › Supplementary_Materials.pdf]

Supplementary Material

1.1 Supplementary Figures

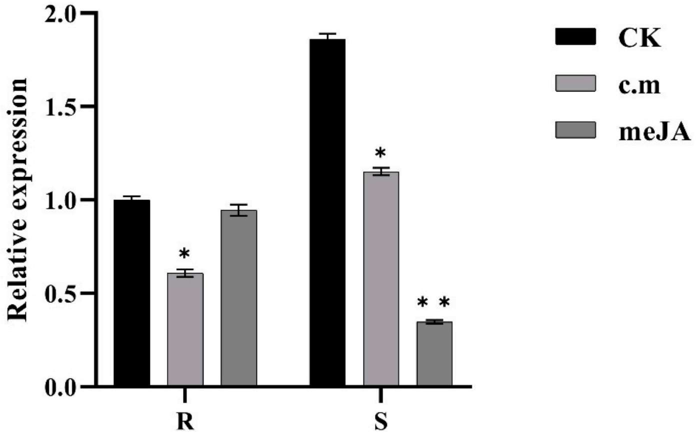

Supplementary Fig. S1. *OsWRKY13* expression in rice leaves infected with *C. m*iyabeanus. **$p <$ 0.01; *$p < 0.05$.

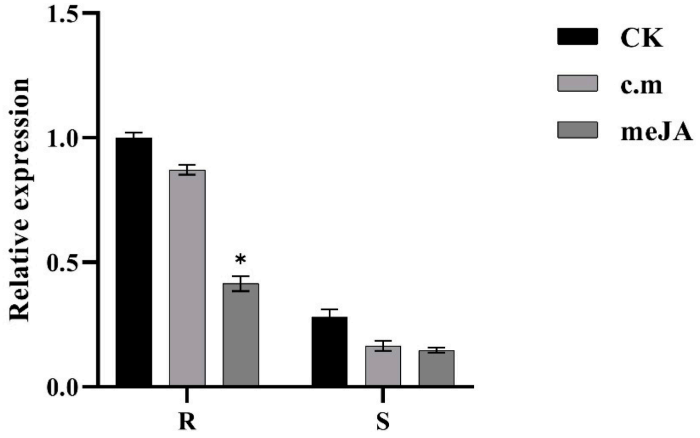

Supplementary Fig. S2. *OsWRKY30* expression in rice leaves infected with *C. m*iyabeanus. ***p* < 0.01; **p* < 0.05.

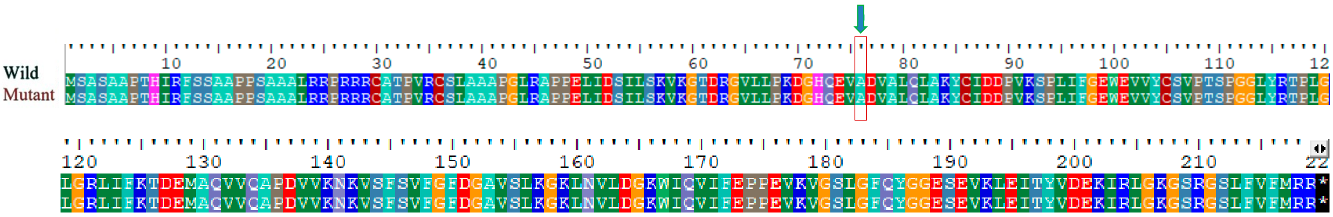

Supplementary Fig. S3. Alignment of OsFBN6 between wild-type and mutant lines; there was no mutation in amino acid Ala at the 76th position.

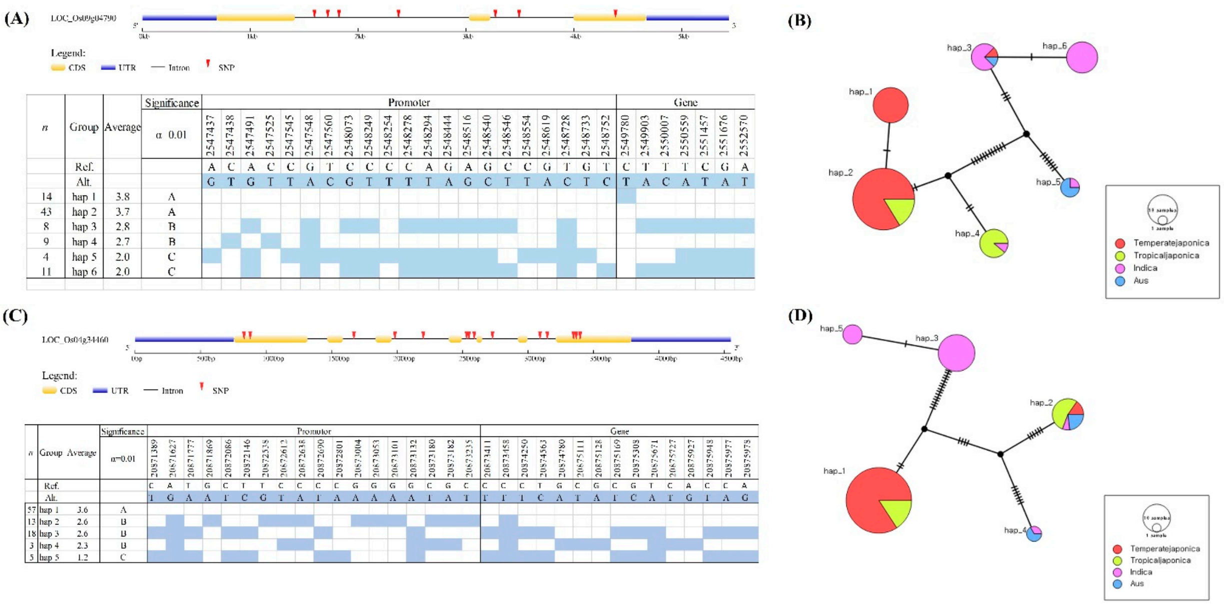

Supplementary Fig. S4. Haplotype analysis. (A, C) Schematic of gene structure and SNP positions in *OsFBN1* and *OsFBN5*. Red arrows indicate SNP sites in the gene region. The average indicates the mean of BS infection scores of all varieties in each haplotype. The subset for α = 0.01 represents a very significant level of difference average of infection in each group. The blue squares represent mutant SNPS in each haplotype (B, D) Haplotype network analysis in *OsFBN1* and *OsFBN5*. Different colors indicate each rice subspecies, and the circle size represents the number of varieties in each haplotype.
